# Three-Dimensional Cell Culture Systems in Pediatric and Adult Brain Tumor Precision Medicine

**DOI:** 10.3390/cancers14235972

**Published:** 2022-12-02

**Authors:** Nicole C. Riedel, Flavia W. de Faria, Amelie Alfert, Jan M. Bruder, Kornelius Kerl

**Affiliations:** 1 Department of Pediatric Hematology and Oncology, University Children’s Hospital Münster, 48149 Münster, Germany; 2 Department for Cell and Developmental Biology, Max Planck Institute for molecular Biomedicine, 48148 Münster, Germany

**Keywords:** organoids, brain tumors, precision medicine, pediatric, glioblastoma, cellular heterogeneity, tumor microenvironment

## Abstract

**Simple Summary:**

High-grade brain tumors, including glioblastoma, are still incurable diseases. Extensive research has allowed new insights into tumor biology, for example, by exploring tumor–TME interactions or their mutational load. Recent advances in in vitro modeling of brain tumors have resulted in the establishment of three-dimensional brain tumor organoid models that recapitulate parenteral tumor characteristics in a more precise manner. This review summarizes the available organoid models in adult and pediatric brain tumors, their limitations, and their present applications. Furthermore, it focuses on their future potential for improving translational therapies based on a better understanding of the molecular bases of tumor biology. We provide researchers with an overview of the field, allowing them to choose a suitable model.

**Abstract:**

Primary brain tumors often possess a high intra- and intertumoral heterogeneity, which fosters insufficient treatment response for high-grade neoplasms, leading to a dismal prognosis. Recent years have seen the emergence of patient-specific three-dimensional in vitro models, including organoids. They can mimic primary parenteral tumors more closely in their histological, transcriptional, and mutational characteristics, thus approximating their intratumoral heterogeneity better. These models have been established for entities including glioblastoma and medulloblastoma. They have proven themselves to be reliable platforms for studying tumor generation, tumor–TME interactions, and prediction of patient-specific responses to establish treatment regimens and new personalized therapeutics. In this review, we outline current 3D cell culture models for adult and pediatric brain tumors, explore their current limitations, and summarize their applications in precision oncology.

## 1. Introduction

Malignant brain neoplasms are a heterogeneous group of tumors, including glioma, ependymoma, embryonal tumors, and many other (rare) entities and subentities, affecting patients from birth to adulthood. Despite intensive treatment protocols, including surgery, chemotherapy, and radiotherapy, the prognosis for many high-grade brain tumor patients remains poor [1,2,3,4,5,6]. Although extensive research in this field has resulted in a detailed molecular classification of brain tumors and led to various new insights into their biology, numerous recent clinical trials have failed to significantly improve the prognosis for these patients, especially those suffering a relapse [7]. Overall, there is a noticeable gap between recent preclinical achievements and the clinical improvements in patient outcomes. This dichotomy may stem from current preclinical studies frequently being conducted in two-dimensional (2D) cell culture, which neither sufficiently recapitulates inter- nor intratumoral heterogeneity nor the cellular diversity of the tumor microenvironment (TME) [8]. Importantly, heterogeneous in vivo-like tumor cell populations respond differently to drug treatment than 2D in vitro entities, and intratumoral diversity results in a higher risk of treatment resistance and tumor recurrence [9,10]. Furthermore, the interplay of tumor cells with their TME, including endothelial cells, immune cells, and neuronal cells, affects the treatment response [11,12]. Therefore, it has become paramount to establish models which include these key characteristics and allow tumor–TME interactions, thus potentially generating more accurate predictions of tumor biology, drug efficacy, and immune response.

In the past few years, organoids emerged as three-dimensional (3D) cell culture systems for modeling healthy and diseased tissues. These organoids potentially model development, diseases, and drug responses [13]. They are self-organizing three-dimensional structures that closely mimic an organ or tissue at a morphological, cellular, and functional level. They can be divided into two major groups: organoids that mirror healthy tissue, including brain organoids, and those that simulate diseased tissue, including tumor organoids [13]. Initial organoid models utilized the intrinsic self-patterning abilities of human pluripotent stem cells in appropriate conditions to generate small aggregates with optic cups [14] or even tissues representing a wide gamut of brain regions, forming the so-called cerebral organoids [15]. Later on, other groups investigated organoids resembling specific brain regions, including the forebrain, the midbrain, the hypothalamus, or the cerebellum [16,17,18]. Pioneering the use of organoids for tumor research, Sato et al. generated 3D in vitro models from primary colon carcinoma samples [19]. They were followed by other groups who developed tumor organoids from various entities, including prostatic, pancreatic, and liver cancers, as well as glioblastoma [20,21,22,23].

These tumor organoids can then be used for high-throughput drug and toxicity screenings to uncover new personalized therapeutics [13,24,25,26]. Additionally, healthy tissue 3D cell culture systems, such as organoids or organ-on-a-chip models, can be further used to test the side-effects of drugs. These technologies may identify drugs with a high efficacy against the tumor and a low burden of side effects on healthy tissues [24,27,28]. Recapitulating parental tumors, cancer organoids have proven to be capable of predicting cancer treatment efficiency in vivo [23,29,30], thus ringing in the era of organoid-based in vitro anti-cancer drug tests.

This review summarizes recent achievements in three-dimensional disease modeling for pediatric and adult brain tumors. Our text is structured in four parts: (I) advantages of 3D over 2D models; (II) current limitations of 3D models; (III) available brain tumor organoid model systems for adult and pediatric brain tumors; and (IV) strategies towards next-generation cancer organoids.

## 2. Organoids Are Superior to Prior 2D In Vitro Models in Recapitulating the Primary Tumor Characteristics

Despite making progress on exploring the mechanisms leading to tumor initiation by identifying, for example, (I) the mutational burden of tumors, (II) malignancies’ cells of origin, and (III) the impact of the TME on tumor cells [31,32,33,34], many key scientific questions to finally improve brain tumor patients’ survival remain unanswered. This lack of fundamental insight may partly stem from in vitro models insufficiently recapitulating the core characteristics of the primary tumors.

**Figure 1:**
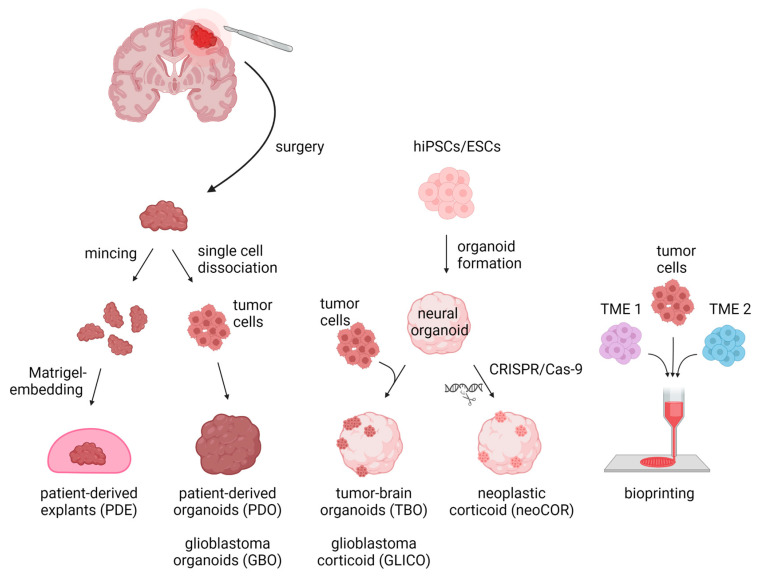
Three-dimensional in vitro tumor models can be derived from either primary patient materials or generated de novo from pluripotent stem cells. PDEs and PDOs are derived from patients’ resected tumor tissue, which is minced and Matrigel-embedded for PDE generation or is single-cell dissociated followed by re-aggregation for PDOs/GBOs. TBOs/GLICOs and neoCORs are generated by seeding hiPSCs/ESCs for organoid generation, followed by co-culture with tumor cells for TBOs/GLICOs or CRISPR-Cas9-based gene editing for neoCORs. To create 3D models via bioprinting, tumor cells are loaded together with TME cells into a bioink and spatially printed. Abbreviations: PDE: patient-derived explant; PDO: patient-derived tumor organoid; GBO; glioblastoma organoid; TBO: tumor-brain organoid; GLICO: glioblastoma corticoid; neoCOR: neoplastic corticoid; 3D: three-dimensional; hiPSC: human induced pluripotent stem cell; ESC: embryonic stem cell; TME1/2: tumor microenvironment cell type 1 or 2. Created with BioRender.com (accessed on 7 November 2022).

For decades, brain tumor research has been based on 2D and 3D cell cultures in mono-layers and spheroids, respectively. Traditional 2D in vitro cultures of tumor models rely on cell propagation in standard petri dishes. These 2D cell culture models undergo clonal selection for fast-growing and cell-culture-compatible cell populations, thereby losing cellular diversity and often resulting in a homogeneous cell population that are no longer recapitulating the tumors’ original heterogeneity [8,35,36]. Due to their mono-layer arrangement, these cell cultures are adapted to conditions of 20% oxygen, which exceeds the usual oxygen level of about 5% in in vivo tumors [8,35]. Spheroids consist of mostly uniform aggregates of a mixture of desired and relevant cell types for a given disease model assembled in an essentially random three-dimensional arrangement. On the other hand, organoids self-arrange their cell types into micro-moieties that more closely approximate organ tissue structure and function. With the advent of these self-organizing organoid tissues, a plethora of more complex three-dimensional model systems have addressed brain tumors in recent years, including patient-derived tumor organoids (PDO), patient-derived explants (PDEs), tumor-brain organoids (TBOs), neoplastic cerebral organoids (neoCORs), and, lastly, bioprinted tumor models (Figure 1). These organoid models can be divided into two major groups: (I) tumoroids starting only from a tumor tissue, including PDOs and PDEs, and (II) organoids composed of a tumor and a non-tumor compartment, including TBOs and neoCORs.

In contrast to traditional mono-layer and spheroid cultures, organoids across all models can preserve intra- and intertumoral heterogeneity [23,29,37] and establish diffusion-limited oxygen gradients within the organoids similar to that in early primary tumors, which may aid in maintaining a diverse tumor cell pool [23,38].

This might be a significant advantage in drug screens, as intratumoral heterogeneity plays an important role in intrinsic and acquired therapy resistance [39]. Two-dimensional cell lines, spheroids, and organoids respond differently to treatment, with organoids more accurately recapitulating the biological response of the parenteral tumor [8,40,41,42]. These findings are driving the hopes for organoid-based drug screens for improved clinical relevance [23,29,40,43].

## 3. Current Limitations of Organoids

**Figure 2:**
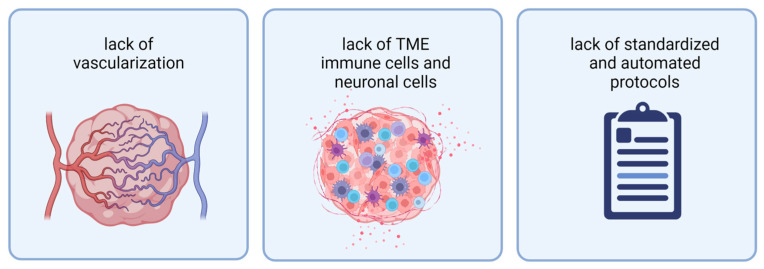
Current organoids face three limitations: from left to right: lack of vascularization, lack of TME immune cells and neuronal cells, and lack of standardized and automated protocols for organoid generation. Abbreviations: TME: tumor microenvironment. Created with BioRender.com (accessed on 7 November 2022).

While 3D model systems have improved over recent years, they still face several limitations. To properly mimic a tumor in vitro, current organoid models are missing vascularization, TME cells (immune cells and neuronal TME), and protocols resulting in more reproducible and scalable organoid generation (Figure 2).

In current brain tumor organoid models, the lack of vascularization impairs their growth, further tumor development, and long-term culture [44]. In brain tumors, the vasculature and blood–brain barrier (BBB) play an essential role in metastasis and selective drug delivery [45]. Despite recent achievements in modeling a BBB in cerebral organoids, vasculature has yet to be introduced into 3D brain tumor models, and this challenge is a very active field of research [46,47,48].

In recent years, several groups have started elucidating the essential role of the TME in brain tumor progression, immune escape, and chemoresistance [11,12,49,50,51]. Therefore, in vitro models should ideally recapitulate the rich gamut of cells, extracellular matrix, and signaling of the TME. However, current organoid models only host single TME cell populations. Primary tissue-derived tumor organoids can retain tumor-resident immune cells for a short time but progressively lose them [29,37]. Recent insights, especially into the importance of neuronal activity and neuron–glioma interactions in glioma proliferation, highlight the need for a neuronal TME in brain tumor models [51]. In TBOs and neoCORs, which are based on cerebral organoids, tumor cells interact with their neural surrounding [40,52,53,54].

Despite their advantages as the next-generation in vitro models, both TBOs and neoCORs are derived from cerebral organoids and, thus, share their challenges. They are difficult to standardize, with a high variance from one sample to the next, likely due to their reliance on self-organization. Currently, the field needs comprehensive strategies to incorporate key cell types, including microglia and near-native levels of astrocytes. These organoids self-arrest at the fetal levels of cellular maturity, peaking at the equivalent of weeks 17–24 of pregnancy [55,56]. Many researchers believe that further maturation requires a functional, perfusable vascular bed and a blood–brain barrier, which has not been demonstrated yet [57]. In this manner, organoids mimic early embryonal brain development (and thus a basic TME) and not mature brain tissue [58].

To date, the variety of models for glioblastoma, including PDOs, PDEs, TBOs, neoCORs, and bioprinting, confronts researchers with the task of choosing the right one for their scientific question. Therefore, we summarize the available models for glioblastoma, low-grade glioma (LGG), and pediatric brain tumors in this review. We further emphasize their distinct advantages for specific applications to facilitate the choice of model, depending on the current research question.

## 4. Glioblastoma 3D Models as Blueprints for Tumor Organoids

Glioblastomas (GBM) are the most common malignant brain tumors in adults and have a dismal prognosis [7,11,49]. They are characterized by extensive infiltration of the surrounding brain tissue, promoted by microtubular networks, which makes them challenging to resect [59]. Moreover, they possess a high intratumoral heterogeneity associated with genetic amplifications driving specific transcriptionally defined subpopulations [60,61].

### 4.1. Glioblastoma Organoids

The most common 3D GBM model are glioblastoma organoids (GBOs). In a group of pioneering studies, these were derived from patients’ primary tissue, minced into small pieces or further enzymatically digested into single cells (Figure 1) [23,29,62]. Furthermore, the resulting small pieces were supplied to a suspension organoid culture (Figure 1) [29]. Alternatively, the single cells were embedded into Matrigel droplets (Figure 1) [23]. In this manner, the organoids grew to a size of up to 4 mm and maintained intratumoral heterogeneity, spatial distribution, as well as hypoxic and stem cell gradients [23]. In addition to glioma organoids, LeBlanc et al. used a similar approach with the so-called patient-derived explants (PDEs) [37]. PDEs were generated by cutting tissue from the margin of both primary and recurrent tumors, dividing it into 1 mm^3^ pieces and placing it into Matrigel (Figure 1) [37]. The passaging of PDEs was performed every 3–7 weeks when tumor cells invaded the Matrigel [37].

Moreover, GBOs can be established and are ready for treatment tests after 2–4 weeks in culture [29,43] or frozen and biobanked for later tests [29]. Marker gene expression for different cell types is consistent over time and after biobanking [29]. The generation of organoids from primary glioblastoma and recurrent tumors has been conducted with a high success rate of about 90% [29]. Besides primary brain tumors, it is also possible to generate PDOs from primary brain metastasis tissue, which typically fails to survive in 2D cell cultures [23]. Thus, these organoids provide additional opportunities for further in vitro research in this field.

In recent years, different techniques, including single-cell RNA sequencing (scRNAseq), have given important insights into transcriptional intra- and intertumoral heterogeneity [60,63,64]. Recently, Neftel et al. described the transcriptional heterogeneity of glioblastoma in four meta-states of neural progenitor-like, oligodendrocyte progenitor-like, astrocyte-like, and mesenchymal-like tumor cells [60]. Compared to 2D cell culture, in which only one or two of these meta-states were recapitulated, the PDEs recapitulated all meta-states present in the primary tumor [37]. The cellular heterogeneity of tumor cells in tumoroids and the related primary tumor correlated well. In contrast, the tumor cells of tumoroids from distinct patients were transcriptionally different, emphasizing intertumoral heterogeneity. Of note, different types of TME cells, including immune cells, endothelial cells, astrocytes, or fibroblasts in the tumoroids, conserved transcriptional similarity across patients [29,37]. However, these non-malignant cells were propagated only in the first-generation PDEs, progressively diminished over time, and were no longer present in later generations [37]. In comparison, glioblastoma spheroids from the same patients did not contain any ancillary cells, neither immune cells nor other cells of the TME [37].

Additionally, GBOs have been used to test distinct treatment modalities, from radiotherapy [23] to chemotherapy [29,43] and innovative targeted therapy [29,43]. In these screenings, organoids serve as the patients’ avatars. In a proof-of-principle study, patients’ outcomes correlated with the organoid response for standard-of-care treatment, consisting of irradiation and temozolomide administration. Thus, patients whose organoids responded to the treatment with a diminishing population of cycling cells showed better clinical treatment responses than the organoid non-responders [29]. Having identified potential targets based on the tumor’s mutations, Chen et al. went one step further and chose the drug for each patient’s treatment based on the organoid response [43]. Firstly, patient-derived GBOs were generated while the patients received standard-of-care treatment. Secondly, when standard-of-care treatment was not effective, the mutational status of the tumor was evaluated, and drugs targeting the mutation were screened on the GBOs. The most effective treatment was applied to the patient. In three reported cases, all patients went into remission [43]. These approaches might display an essential link of in vitro testing to clinical applications but need further examination as both cited studies only included a limited number of patients. Overall, organoid-based drug screenings might be a promising avenue towards precision oncology in brain tumor patients.

In addition to evaluating standard drugs, including chemotherapy and/or small compounds, GBOs can be used for pre-clinical tests of CAR-T-cell therapy [29]. In previous research, co-culturing GBOs with CAR-T-cells was performed for 4–8 days in CAR-T-cell medium. GBOs tolerated this medium and reacted with diminished proliferation [65]. After the CAR-T-cell invasion into the organoids, the EGFR-positive tumor cells were successfully killed, while the EGFR-negative tumor cells survived treatment [29]. Here, organoids with intratumoral heterogeneity of gene expression, which included EGFR, showed a significant advantage compared to experiments in 2D cell cultures, where the targets are artificially overexpressed in all cells [29,66].

In contrast to GBOs, spheroids are arguably easier to standardize and scale-up, and they allow the generation of results with a lower variance, which is potentially more suitable for screening. Thus, scientists seeking the most comprehensive GBM model with a complex TME are directed towards organoids and PDEs (if immune cell presence is essential), and researchers looking to implement larger screens are directed towards spheroids. Their short in vitro generation period of only two weeks makes tumoroids especially suitable for fast tests for personalized precision oncology. Furthermore, no special equipment or additional cells are needed for tumor organoid generation, so it might be a model that is easier to establish than more complex embryonal stem cells (ESCs) or human-induced pluripotent stem cell (hiPSC) derived models, including neoCORs and GLICOs.

### 4.2. Glioblastoma Corticoids

Glioblastoma corticoids (GLICOs) have been extensively studied since cerebral organoids emerged in the scientific community [15,40,54,67,68,69,70]. Da Silva et al. investigated a co-culture of 12-day-old murine cerebral organoids with GFP-labeled glioblastoma spheroids to test tumor cell invasiveness. After 48 h of co-culture, immunofluorescence imaging revealed that the glioblastoma stem cells (GSCs) invaded the core of the organoids [67]. Due to the slower maturation of human neural organoids, other groups studied the invasiveness of GSCs in human cerebral organoids derived from pluripotent stem cells at 24 days or 30 days of age from the start of the co-culture [54,69]. The number of invading cells per organoid decreased with the age of the organoid after co-culture started [69]. Furthermore, GBM cells from relapsed patients showed a more invasive pattern, characterized by microtubules promoting the invasion into the core of the organoids [69].

The crucial role of neuronal TME cells in GBM was first described by Venkatesh et al., who found that glioma growth was promoted by neuronal activity-related neuroligin-3 release, which correlated with an inferior prognosis [50]. Moreover, glioma cells were integrated into neuronal circuits through neuron-glioma synapses and were electrically coupled. In this manner, neuronal activity in the neuron-glioma synapses promoted tumor proliferation [51]. Hence, tumor-brain organoids (TBOs), harboring a malignant and a non-malignant compartment, present an innovative and promising opportunity for further research in this field. Another aspect is that glioblastoma cells are connected through a microtubule network, which makes them resistant to radiotherapy due to their ability to rapidly repopulate from the remaining cells in the microtubule network [71]. The characteristic tumor-microtubule formation has also been observed in GLICOs, forming a network that promotes invasion [40,54,69]. A screening revealed that GBM cells in GLICOs were more resistant to chemotherapy and radiation when compared to 2D cell cultures [40], possibly due to the microtubular network. Interestingly, Linkous et al. observed cytoplasmatic fusions between different glioma cells and between glioma cells and neurons in GLICOs [40]. This, again, stresses the importance of including the neuronal microenvironment in glioma models.

**Figure 3:**
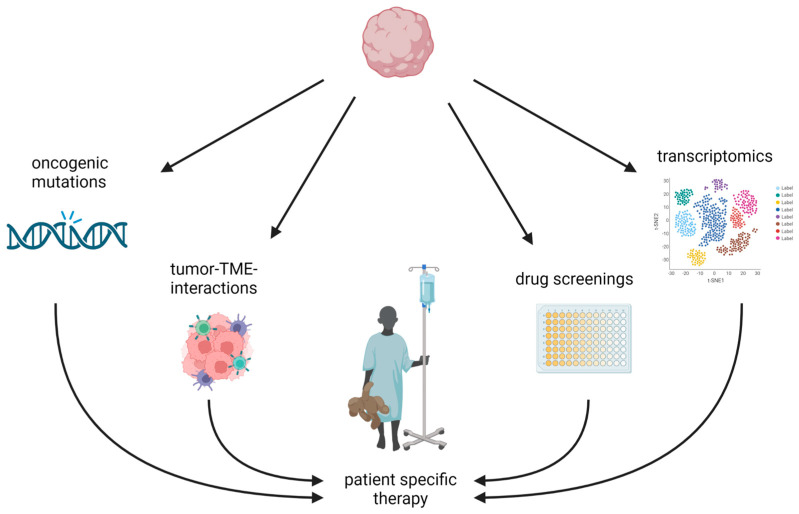
Broad possibilities for organoid applications pave the way to precision medicine. From left to right, latest technologies help uncover neurodevelopmental effects of oncogenic mutations, tumor–TME interactions, drug screenings, and transcriptomics and can drive the development of patient-specific therapies. Abbreviations: TME: tumor microenvironment. Created with BioRender.com (accessed on 7 November 2022).

In summary, by integrating tumor cells into a (human) neuronal surrounding, GLICOs are a promising new model as microtubule networks are essential for tumor invasion and resistance [51,71]. Using human brain organoids might be advantageous as human-specific brain developmental features are not recapitulated in murine brain organoids [15]. One promising possibility for future use of these organoids is addressing the neuronal–tumor interactions in this system at both the functional and the transcriptional levels (Figure 3). Generally, scRNAseq of organoids may, in the future, uncover tumor–neuronal–TME interactions at a transcriptional level and identify transcriptional programs, especially in therapy-resistant tumor cells, that may yield new targets for precision medicine. In addition, spatial transcriptomics, which retains the anatomical information of transcriptomic features, may map tumor cells in GLICOs and at the same time determine the possible characteristics of more invading or resident tumor cells. These results may later help to target a spatially distinct tumor population that has emerged as being more aggressive or therapy-resistant. Next to more basic research, GLICOs show advantages over other systems in drug screenings, as they combine both neuronal and malignant tissues, which might enable researchers to screen for neuronal and cancer toxicities simultaneously.

### 4.3. Neoplastic Cerebral Organoids (neoCORs)

A decade ago, CRISPR-Cas9-mediated genome editing emerged as a reliable and precise method to insert a specific gene sequence into the genome [72]. Since then, CRISPR-Cas9 technology has been used for genetic modifications to explore oncogenic mutations driving tumor progression and metastasis in vivo and in vitro [73,74]. Genetic modifications in cerebral organoids can uncover the mechanisms of tumorigenesis by selectively modifying specific genes and thereby revealing their (dys-)function in brain neoplasms. The so-called neoplastic cerebral organoids (neoCORs) leverage CRISPR-Cas9-based gene editing to either introduce oncogenic mutations or induce oncogene expression in the developing organoid (Figure 1) [53,75]. Using this approach, only a subset of cells expresses the mutation while the majority remains unmodified. This mirrors in vivo tumor formation, where mutated cells transform into tumor cells in a surrounding of non-malignant cells. In a TP53-mutated model of neoCORs, Ogawa et al. observed that the mutated cells proliferated rapidly, overgrowing the rest of the organoid tissue [75]. Transplanting these organoids into mice confirmed this invasive and aggressive phenotype in vivo [75].

In a different approach, Bian et al. screened for the oncogenic capacity of various amplifications or mutations by introducing them together with green fluorescent protein (GFP) as a marker via CRISPR-Cas9 in pre-differentiated organoids at the end of the neuronal induction period [53]. They screened for mutations or amplifications, alone or in the clinically most relevant combinations, associated with GBM, medulloblastoma, atypical teratoid and rhabdoid tumor (ATRT), and other rare tumors. None of the single mutations and just a few combinations resulted in an overgrowth of tumor cells over the rest of the organoid [53]. As one of the combinations resulted in an EGFR-overexpression, they tested anti-EGFR targeted therapy on the resulting organoids. This led to a shrinkage of the tumor population compared to the vehicle-treated organoids [53]. Thus, neoCORs may elucidate the effect of tumorigenic mutations in a developing brain by mimicking the early stages of tumorigenesis, opening this field for future research. However, single- or multiple mutations found in glioblastoma patients do not automatically lead to tumor development in organoids [53]. These results underline the complexity of tumor development, which still can only be partially recapitulated in current organoid models.

Further investigations into adapting these models are required to overcome these limitations. As neoCORs mimic tumor development in a neuronal surrounding, investigations in how the TME plays a role in tumor maintenance, progression, and tumorigenesis might lead to new biological insights into the pathways involved. In addition, these organoids might be a suitable in vitro platform to test new drugs for specific mutations while simultaneously assessing their effect on the surrounding healthy neuronal microenvironment.

In summary, neoplastic cerebral organoids will find their applications in research addressing tumor genetic modifications involved in malignancy initiation, proliferation, and metastasis. NeoCORs can also uncover the different effects of these mutations on different cell types or states in the developing brain, possibly pointing to candidates for the tumors’ cells of origin. In the future, these insights may open new opportunities for custom-targeting strategies against tumor-specific mutations, for example, via gene therapy.

### 4.4. Bioprinting

Three-dimensional tumor modeling can be performed not only in organoids but in bioprinted cellular aggregates, as first achieved in glioblastoma [76,77,78]. In general, bioprinting uses several distinct cell types suspended in hydrogel-based bioinks. These bioinks can be assembled into complex, customizable 3D structures in a layer-by-layer strategy [79]. One significant advantage of this technique is that diverse cell types can be composed in known proportions and spatial configurations, potentially mimicking tumors and their microenvironment. However, despite the significant advances in a fast-growing field, the spatial resolution, reproducibility, and scalability are still limited, specialized formulations of ECM and media are needed, and the printing process is time-consuming, costly, and potentially harsh/not suitable for all cell types [80,81].

Yi et al. used patient-derived tumor cells, endothelial cells, and ECM components to recapitulate the spatial composition of a tumor [78]. Utilizing this technology, a bioprinted tumor-on-a-chip model can recapitulate patient-specific treatment responses, such as resistance to chemoradiation and temozolomide [78]. In addition to the neuronal and vascular TME, another key factor for anti- or pro-tumoral activity is the presence of immune cells. Glioma-associated macrophages (GAMs) play an essential role in tumor progression, invasion, neoangiogenesis, and shaping of an immunosuppressive TME [82]. In bioprinted models, GBM cells can be printed together with macrophages. Resembling in vivo interactions, tumor cells support a GAM phenotype formation. Subsequently, GAMs promote invasiveness, progression, and changes in the treatment response of the tumor cells [76,77]. Furthermore, it is possible to perform bioprinting in 96-well plates, which makes it suitable for future drug screens [83].

Bioprinting has emerged in glioblastoma research as a new opportunity to study tumor–TME interactions in a spatial and scalable manner. Special equipment and knowledge needed for bioprinting complicates the use of this technique. Although bioprinting GBM models have yet to prove their ability to recreate the tumor or brain’s histological, transcriptional, and functional features, they can integrate a variety of distinct cell types in a spatially controlled manner. In this way, future bioprinted GBM models can enable researchers to study tumoral interactions with their microenvironment and the resulting downstream effects on progression, invasiveness, and treatment resistance.

### 4.5. Generation of Patient-Derived Organoids from Lower-Grade Glioma

In contrast to the organoid generation from high-grade glioma [29,40,54], the generation of organoids from low-grade glioma (LGG) remained unaddressed for a long time. However, finding new methods to generate patient-specific in vitro models is vital as the establishment of cell lines from LGG patients in 2D has mostly failed [84]. Recently, the generation of organoids from LGG succeeded [84]: organoids were generated by cutting the primary tumor tissue into 1 mm^3^ pieces, followed by suspension culture on an orbital shaker. Hypothesizing that the tumor might need an oxygen level similar to the in vivo conditions, 21% versus 5% oxygen levels were tested in the culture. In contrast to the 21% oxygen levels, a normoxic physiological tumor oxygen level of 5% led to more intact organoid tissues and an increased organoid diameter [84]. Moreover, LGG-organoids recapitulated parenteral tumor characteristics, including mutational burden, cytoarchitecture, number of proliferating cells, and the presence of macrophages, microglia, and vascular cell populations [84]. Thus, current methods for organoid generation could be applied to other brain tumor types or subgroups with minor adaptations in the culture conditions to address the specific requirements of each entity. Especially for LGG, organoid generation is a significant step forward in providing sufficient high-quality source material for in vitro studies of a tissue that could not be maintained ex vivo before.

## 5. Organoid Models in Pediatric Brain Tumors

Pediatric brain tumors are much rarer than brain tumors in adults but belong to the most frequent tumor entities in children. Besides pediatric glioma and ependymoma, children are affected by embryonal tumors, including medulloblastoma, atypical teratoid and rhabdoid tumor (ATRT), and embryonal tumor with multilayered rosettes (ETMR), most of which are associated with a poor prognosis [85]. Despite recent achievements in establishing organoid models for adult brain tumors, such as glioblastoma or LGG [29,40,54,84], a similar body of work for pediatric brain tumors is still missing (Table 1). Unfortunately, the results gathered from in vitro tumor models cannot necessarily be transferred from adult to pediatric entities as tumor location and molecular characteristics of the tumor and the developmental state of the brain, as a key player of the TME, differ. Recent studies have shown that drugs that work perfectly in adults may cause major side effects on the developing brain of children [86]. Nevertheless, drugs are primarily established for adults and then transferred to pediatric patients [86]. Using 3D cell culture techniques might help to overcome this problem and directly establish drugs for this age group. Thus, one major challenge is the development of individual model systems for each pediatric brain tumor entity and subentity. In the last few years, some groups started testing PDO or neoCOR models for some of these entities [52,87,88,89]. Due to the scarcity of publications, the next section will summarize recent achievements by entity and not by model.

### 5.1. Pediatric High-Grade Glioma

Pediatric high-grade gliomas (HGGs) share many features of HGGs in adults but are genetically different. Therefore, models addressing the specialties of this age group merit their own disease models [90]. Recently, Sundar et al. generated organoids from pediatric HGG patients [87]. Here, organoids were formed by embedding single cells into Matrigel, followed by a shaking culture [87]. Distinct proliferative phenotypes were observed in the organoids pre- and post-treatment and evaluated via immunohistochemistry microarrays of the organoids. By testing the effects of the clinical standard of care (temozolomide and radiotherapy) on the proliferation of glioma sphere cultures and organoids, Sundar et al. found the organoids were resistant to this therapy, while the glioma spheroids stayed sensitive [87].

### 5.2. Medulloblastoma

Medulloblastoma is the most frequent malignant brain tumor of the cerebellum in children [91] and is divided into four consensus molecular subgroups: WNT, SHH, Group 3, and Group 4 [92]. Despite recent progress in the establishment of in vitro and in vivo medulloblastoma models, current models mainly cover SHH and Group 3 medulloblastoma and models for WNT and Group 4 are clearly underrepresented [93,94]. Therefore, a majority of patient tumors are not represented in current preclinical studies and the resulting information might only be relevant for a small group of patients [94].

Ivanov et al. pioneered a co-culture model of neuronal stem cells with tumor cells in spheroids to mimic tumor–host interactions for a cytotoxicity screen [42]. They generated medulloblastoma-neuronal stem cell (NSC) spheroids by seeding the same amount of medulloblastoma cells and NSCs, both labeled with distinct fluorescent dyes, followed by seven days of culture. For the cytotoxicity screen, spheroids were treated with different concentrations of Etoposide on day 3. Due to the presence of both tumor and healthy stem cells in the spheroid, they were able to simultaneously assess the toxicity in both compartments by dissociation, followed by flow cytometry on day 7 [42]. Later, other groups used organoids to model medulloblastoma in vitro, using either organoids derived from patient cells [89] or neoplastic cerebellar organoids [88]. Medulloblastoma organoids can be derived from single cells and similarly cultured in Matrigel as glioblastoma organoids [23,89]. Ballabio et al. used another approach: they first pre-differentiated cerebellar organoids until day 35, when all progenitors were present, and this was followed by transfection of the intact organoid with several potential oncogenic mutations derived from a patient-specific screening [88]. This approach proved the ability of Oct-2 and c-Myc mutations to elicit a medulloblastoma-like phenotype in the organoids [88]. These pioneering studies display an important starting point of 3D medulloblastoma modeling which might help establish model systems that can represent the whole molecular diversity of medulloblastoma, via direct patient-derived organoids, or help establish organoid modeling for all entities in future studies.

### 5.3. Atypical Teratoid Rhabdoid Tumors

ATRTs, belonging to the group of embryonal brain tumors, are much less prevalent than medulloblastoma. ATRTs are characterized by the loss of the SMARCB1 or SMARCA4 gene, which encodes for a subunit of the SWI/SNF chromatin-remodeling complex [52,95]. Through the inactivation of SMARCB1 with CRISPR-Cas9 in neoCORs, Parisian et al. uncovered the effects of its knockdown (KD) on neuronal development [52]. The impact of SMARCB1 KD on the cells depended on the organoids’ developmental stage. Interestingly, the KD blocked differentiation termination only in neuronal progenitor cells, while mature neuroblasts stayed unaffected, and human-induced pluripotent stem cells (hiPSCs) died [52]. This might indicate that malignancy initiation is only possible in a specific developmental window. In this model, only early neuronal progenitor cells had the capacity to transform into tumor cells [52], which hinted at the progenitor cells being the potential cells of origin for this tumor entity. This finding in organoids recapitulates current findings in mice, in which only defined cells of origin and differentiation states give rise to ATRT development when Smarcb1 or Smarca4 is lost [31,96,97,98].

### 5.4. Conclusions for 3D Models of Pediatric Brain Tumors

In summary, few organoid models have been established for pediatric brain tumors, and published work only exists for a subset of the overall tumor entities [52,87,88,89]. This may be partially due to the overall rarity of these diseases, and, thereby, limited sample availability. Examples of organoid models in pediatric brain tumors have shown that the same techniques used for adult glioblastoma can be transferred to pediatric brain tumors, opening similar opportunities for a wide range of potential applications in cancer research and personalized medicine. Special difficulties include how to model the variety of different entities and associated microenvironments. Differences in the componence of the tumor microenvironment, especially in the immune microenvironment, need to be considered when designing and establishing a new model. For instance, the immune infiltration in embryonal tumors, such as ATRT, medulloblastoma, and ETMR, is much lower than that of gliomas [99]. Additionally, within one entity, such as medulloblastoma, the infiltrating immune cell populations and numbers are significantly differing between distinct subgroups [99]. This makes the development of 3D models, which include also immune cells, even more challenging. Moreover, in this field, further research is needed to establish organoid models for entities that have not yet been addressed, including ependymoma and ETMR.

## 6. Generation of Next-Generation Organoids via Vascularization, Integration of TME Cell Diversity, and Standardization/Automation

### 6.1. Vascularization and Blood–Brain Barrier in Current Brain Organoid Models

Several groups have recently approached vascularizing brain organoids [46,47,48]. One of the first methods was to embed pre-formed organoids with hiPSC-derived endothelial cells in Matrigel [46]. Notably, this approach resulted in an infiltration of endothelial cells in the outer organoid cell layers [46]. In a different approach, Shi et al. co-aggregated hiPSC cells and human umbilical vein endothelial cells (HUVECs) to promote potentially vascularized organoid formation [47]. When transplanted into mouse brains, the vascularized organoids connected to the murine vessels and were perfused [46,47]. Besides endothelial cells, smooth muscle cells (SMC) or pericytes are necessary for blood vessel formation. Via the integration of SMC and endothelial cells, which were differentiated from mesodermal progenitor cells, it was possible to generate blood vessels in the organoid. Under hypoxic conditions, vasculogenesis was promoted, and the cells were able to build a vascular network spreading through the entire organoid [100].

The first forays into complex vascularization are emerging in the literature. Ahn et al. and Sun et al. demonstrated comprehensive vascularization by a co-culture of blood-vessel organoids with cerebral organoids, with the presence of most of the blood–brain barrier (BBB) components, including SMC, pericytes, endothelial cells, and the basement membrane, and only lacking astrocytes [48,101]. The resulting organoids had an increased pool of neuronal progenitor cells [101], and increased neurogenesis [102,103]. Furthermore, the embedded vascular network recapitulated some BBB-markers, including α-ZO1 [48,103]. However, to date, efficient bulk perfusion of organoid tissues from self-organized vessels has not been demonstrated yet. For cancer vasculature modeling, some co-culture models of cancer organoids with different vascular models have also been used in the past [104,105]. In a co-culture of breast cancer organoids and tissue-engineered microvessels, the tumor cells invaded the endothelial vessel wall and formed mosaic vessels, as observed previously in mouse models [105]. In a co-culture of tumor organoids with perfused vasculature-on-a-chip models, tumor cells were able to invade into the vessels and this system could be used for drug tests [104].

These results indicate that vascularization of brain tumor organoids is, in principle, feasible, although much work remains. The characteristic features of the BBB and the vasculature change in brain tumors result in more irregular tumor vasculature and a blood–brain barrier with higher permeability, also known as the blood–tumor barrier [106]. This vasculature needs to recapitulate tumor-vasculature-specific (dys-)functions after vascularization in brain tumor organoids. The current literature suggests that vascularized organoids in the future could be promising strategies to investigate tumor–BBB interactions and to model changes in the vasculature and BBB towards a tumor-specific phenotype [44]. Moreover, this approach may help emulate the BBB for drug delivery approaches in vitro, as the BBB is a formidable barrier towards drug delivery.

### 6.2. Tumor Microenvironment

In addition to the vasculature, next-generation brain tumor organoid models should contain all other cell types of the diverse TME, including immune cells, glia, microglia, and various neuronal cells. Several studies added elements of the TME to organoids. For example, to enrich organoids with immune cells, Jacob et al. co-cultured CAR-T-cells with organoids [29,65]. However, Jacob et al. only tested a short-term co-culture of 4–8 days [24]. Long-term culture of different cell types in one organoid still requires the development of a cell culture medium that is suitable for the differentiation, function, and maintenance of all cell types. In brain neoplasms, microglia, the brain-resident macrophages, play an essential role as tumor-associated macrophages by promoting proliferation and invasion [107]. Previously, Xu et al. developed a protocol for integrating microglia in cerebral organoids in a controllable ratio [108]. These microglia then exhibited their natural functions, including synaptic pruning and phagocytosis [108].

In the future, it might be advantageous to use self-organizing complex tissue models, such as organoids, as a basic scaffold to be enriched with the key cells of the TME. Including controlled amounts of microglia and other immune cells can help fine-tune the model to better reflect a tumor-specific immune response in vitro. By overcoming the challenge of incorporating the whole TME diversity in an organoid model, it will be possible to mimic the specific tumor niche more accurately in vitro and, thus, foster the use of organoids as patient avatars in precision medicine.

### 6.3. Standardization and Automation of Organoid Culture

Current in vitro tumor models suffer from several sources of variance that render them difficult to compare across techniques, labs, and even different patients as cell source. Unified methods for generating 2D or 3D models are scarce, which results in methodological differences that change from one lab to another. With different sample and cell line sources and handling histories, even careful quantification of relevant metrics does not necessarily reflect the superiority of one approach over another. Thus, a comprehensive comparison between 2D cell cultures and the benefits of one organoid model over another is still missing. The field as a whole would benefit from larger studies that quantitatively compare several highly standardized models and protocols in their ability to recapitulate the parenteral tumor characteristics. This would help to clarify the reliability of distinct models and the reproducibility of results across different strategies (2D/3D) and tumor models (i.e., PDOs and TBOs).

Another inherent source of variance in organoids is their complex self-organizing biology combined with extensive and variable manual handling, two factors which often underlie significant variations between samples of the same batch and between different batches. Automating organoid culture is a promising approach to increase reproducibility through a more homogeneous and standardized workflow, which also makes the systems scalable for high-throughput screening strategies. Recently, Renner et al. succeeded in fully automating mid- and forebrain organoid generation, culture, and optical analysis [109,110] in a 96-well format. Their automated midbrain organoids were highly homogeneous and reproducible in size, marker gene expression, and transcriptional levels [109]. Furthermore, they established a high-throughput screen that assessed different neuronal subpopulations while retaining their spatial information through automated whole-mount immunostaining, tissue clearing, and image analysis [109,110]. Translating other current 3D brain tumor models to an automated workflow could further increase the homogeneity and reproducibility of cancer organoid approaches. For example, a cell type-specific chemotoxicity screen in brain tumor organoids could evaluate and improve the targeted therapy of treatment-resistant tumor subpopulations in precision medicine.

## 7. Conclusions

Three-dimensional in vitro models, such as organoids, ring in a new era in brain tumor research, opening opportunities for personalized brain tumor precision medicine. Their potential to recapitulate inter- and intratumoral heterogeneity and other characteristics of their parenteral tumor, such as histology, mutational burden, and transcriptional patterns, makes them superior to 2D cell line-based in vitro models. Besides applications in basic research, including mechanisms of tumorigenesis and tumor–TME interactions, they may also serve as patients’ avatars in drug screens and pre-evaluation of targeted therapy approaches before the clinic. However, although present research is promising, current organoid models nonetheless still require functional incorporation of all components of the tumor microenvironment, namely, neuronal, immune, and vascular cells, in order to model the tumor and its TME in its complexity. Although further improvements in scalability, reproducibility, and overall standardization are needed to bring these models to a clinically relevant stage for patient-specific therapies, three-dimensional brain tumor models have already proven reliable in mimicking tumor-specific drug responses. The future will drive more comprehensive, complex, scalable, and reproducible tumor organoid models for an ever-increasing number of tumor entities. This development has true potential to drive innovation at the basic science and at the clinical level, bringing together 3D biology, genetic engineering, patient-derived samples, and new screening strategies for next-generation custom-tailored, patient-specific precision medicine.

## Figures and Tables

**Table 1:** Established three-dimensional brain tumor models.

Reference	Entity	Model-Type	Method
Hubert et al., 2016 [23]	glioblastoma	GBO	Tumor cells embedded in Matrigel
Jacob et al., 2020 [29]	glioblastoma	GBO	Tumor pieces on an orbital shaker
Loong et al., 2020 [62]	glioblastoma	GBO	Tumor cells embedded in Matrigel
Chen et al., 2022 [43]	glioblastoma	GBO	Tumor pieces on an orbital shaker
LeBlanc et al., 2022 [37]	glioblastoma	PDE	Tumor pieces in Matrigel
da Silva et al., 2018 [67]	glioblastoma	GLICO	Murine brain organoids, GBM cells
Linkous et al., 2019 [40]	glioblastoma	GLICO	Brain organoids + GBM cells
Krieger et al., 2020 [54]	glioblastoma	GLICO	Brain organoids + GBM cells
Gorancia-Buzhala et al., 2020 [69]	glioblastoma	GLICO	Brain organoids + GBM cells
Azzarelli et al., 2021 [70]	glioblastoma	GLICO	Brain organoids + GBM cells
Ogawa et al., 2018 [75]	glioblastoma	neoCOR	HRas, TP53 mutations
Bian et al., 2018 [53]	glioblastoma	neoCOR	Several different mutations in combination and alone as PTEN, Myc, and EGFR
Yi et al., 2019 [78]	glioblastoma	bioprinting	GBM cells + endothelial cells + HUVECs
Heinrich et al., 2019 [76]	glioblastoma	bioprinting	GBM cells + macrophages
Tang et al., 2020 [77]	glioblastoma	bioprinting	GBM cells + neuronal progenitor cells + astrocytes + macrophages
Abdullah et al., 2022 [84]	LGG	PDO	Tumor pieces on an orbital shaker; 5% O_2_
Sundar et al., 2022 [87]	pediatric HGG	PDO	Tumor cells embedded in Matrigel
Frisira et al., 2019 [89]	medulloblastoma	PDO	Tumor cells embedded in Matrigel
Ballabio et al., 2020 [88]	medulloblastoma	neoCOR	Different mutations, e.g., Otx-2 or c-Myc
Parisian et al., 2020 [52]	ATRT	neoCOR	SMARCB1-KD
